# The analysis of citation in headlines in the Spanish press

**DOI:** 10.1016/j.heliyon.2019.e03155

**Published:** 2020-01-09

**Authors:** Ekaterina Terentieva, Galina Khimich, Irina Veselova

**Affiliations:** Foreign Languages Department, Faculty of Humanities and Social Sciences, Peoples' Friendship University of Russia (RUDN University), 6 Miklukho-Maklaya Street, Moscow, 117198, Russian Federation

**Keywords:** Spanish mass media, Citation, Reported speech, Headlines, Cultural sociology, Language development, Media sociology, Pedagogy, Sociolinguistics, Sociology, Linguistics

## Abstract

Citation constitutes one of the most important components of text in all mass media, allowing for the concretization of events and serving to enhance media authenticity. The aim of this work is to study the structural features of the citation technique across headlines in the electronic editions of five leading Spanish mass media outlets between 2010 and 2018. Determined for the purpose of the study were the forms and frequency of three types of citation – direct, indirect and mixed – along with the structural features of the methods by which citation is integrated into a newspaper text. The conducted content analysis demonstrates that citation-driven headlines account for 15% of the total processed corpus and also clarifies the prevalence of direct citation (53%) among the three types. The division of source texts into two groups enables us to distinguish implicit citation as a special type tasked with citing the precedent text. In addition, three primary predicate groups are singled out: declarative, explicative and evaluative. These serve to introduce direct and indirect citations. The analysis of the narrating remark highlights the importance of the pragmatic function of citation in a newspaper text. The study confirms the significance and relevance of employing citation in headlines in the Spanish press and provides insight into the phenomenon of reported speech and the function of citation in mass media.

## 1 Introduction

Mass media texts constitute a social phenomenon in the system of social control and play a leading role in employing language as a tool of influence. A specific character of discourse activity within mass communication, an anticipated or planned reaction of the addressee and deliberate distortions of information all give evidence to an emergent function of language itself – control over the behaviour of vast masses of people and manipulation of their consciousness.

Today, information has become a decisive strategic factor across all spheres of human life. Edward Sapir (1993) pointed out that the history of civilization is characterized by constant expansion of the effect scope of communication. The modern person's worldview, to a substantial degree, is composed of knowledge he or she acquires from books, news outlets, radio and TV programmes, and, predominantly, Internet-based sources. By informing people of the global state of affairs and filling up their spare time, mass media outlets impact their entire mindset and style of worldview – and through that – the type of contemporary culture. This contemporary culture is characterized by the immediacy and transience of information, its ideology-driven nature, the rise of specific language and synchronous simplicity of perceiving mass media texts, which nevertheless claim to be the absolute truth (Van Dijk, 1977, 2003; 2006; Vicente Mateu, 2007; Reah, 2002). Citation plays a key role in creating the effect of informational authenticity within the language of mass media.

One of the frequent structural components of a newspaper text is citation, which is widely used in modern published news sources, specifically, because it allows the author to solve a number of issues: to represent certain factual information in a precise manner and indirectly express his or her attitude towards it, thus – in the case of direct citation – simultaneously creating a special presence effect intensifying the impact upon the addressee (Méndez García de Paredes, 1999; Harry, 2013). The existence of direct and indirect quotations is recognized as a constant feature of the functional sphere of a newspaper text. Citation may also be viewed as a mechanism of major importance for delivering coherence and integrity of the textual nexus (Clayman, 1990; Smirnova, 2009).

The extensive use of citation in headlines in the Spanish press and the distinct significance of this element in newspaper texts have become the object of special research.

## 2 Theory

### 2.1 Concerning the “foreign” word in the “native” one

Linguistic studies of today devote considerable attention to the topic of citation, the concept of the "foreign” word in the “native” one (Arutyunova, 1992; Beltrán Almería, 1990; Bruña Cuevas, 1990; Cappelen and Lepore, 2005; Méndez García de Paredes, 2000a; Nadal Palazón, 2008).

The analysis of the above topic notably influenced the utterance concept proposed by Émile Benveniste (1966) and his linguistic theories.

The concept of “foreign” had an impact on the study of polarization in the native and the foreign as a phenomenon; this concept manifested itself in various theories and defined a plethora of directions and notions in modern linguistics. This impact is discernible within the ideas of pragmatics and the speech act theory, as well as within Mikhail Bakhtin's own theory of textual polyphony and those of his school. The above saw further development in the works of Oswald Ducrot (1984).

As we know, the issue of the “foreign” word in the “native” one was formulated by Mikhail Bakhtin in the mid-1930s in relation to a literary text. In his work “Problems of Dostoevsky's Poetics” he stated, “The entire life of language, in any area of its use (in everyday life, in business, scholarship, art, and so forth), is permeated with dialogic relationships” (Bakhtin, 1984: 183).

One of the fundamental works to analyse the problem of reported speech belongs to a scholar from the so-called Bakhtin's circle, Valentin Voloshinov: “Marxism and The Philosophy of Language” (1930). The above work features specific chapters dedicated to the definition of reported speech and a detailed overview of various “syntactical patterns” and their modifications of the way reported speech is conveyed in the Russian language. The author proceeds from the following understanding of the phenomenon in question: “Reported speech is regarded by the speaker as an utterance belonging to someone else, an utterance that was originally totally independent, complete in its construction, and lying outside the given context. Now, it is from this independent existence that reported speech is transposed into an authorial context while retaining its own referential content and at least the rudiments of its own linguistic integrity, its original constructional independence” (Voloshinov, 1973: 116).

Among other things, theoretical questions of citation have been researched by language philosophy works, thoroughly analysed by Donald Davidson (2001).

Since the 1970s–80s, the issue of reported speech has been widely discussed as pertaining to the theory of reference. A question of conveying another person's speech emerges in conjunction with the speaker's reference; one may choose to designate the referent as either the one speaking or as the author of the conveyed statement. Various approaches to defining the significance of referential utterances caused two arrays of similar notions to appear: 1) referential use of names and descriptions – belief *de re*; 2) attributive use of specific descriptions – belief *de dicto* (Arutyunova, 1982).

### 2.2 The citation theory

According to the theory of textual polyphony of Mikhail Bakhtin and his school, assuming the idea of the “foreign” word in the “native” one, we propose to define the general phenomenon of citation as representing a foreign text (source text) within the recipient text. Every element of the foreign text included in the author's text appears to be a citation. Citation is not limited to a verbatim reproduction of an utterance: a citation is any case of an addressee recognizing (or being understood to recognize, having sufficient background knowledge) the intention of the addresser to bring forward another person's utterance. Various aspects of citation have become a subject of numerous studies (Bruña Cuevas, 1993; Maldonado González, 1991; Méndez García de Paredes, 1999, 2000b; Reyes, 1993, 1994; Myasnikov, 2008; Shpilnaya, 2014).

Citing another person's or one's own words in direct or indirect speech, resorting to allusions and appeals to intertextuality, and using another person's vocabulary or intonation are an ever-present phenomenon for any type of speech. By way of citation, the speaker ascribes specific words to another person, whether it be a precise reproduction of another person's utterance, a summary of its content or a combination of both.

Such attribution may be erroneous, should a speaker ascribe to another person what he or she has never said at all or never said in a specific way. It may be approximate, when another person's words are not cited word for word and instead only the meaning of the words is conveyed; it may be a reconstruction of what the other said. Attribution may be fictitious (e.g., when dealing with book characters). Even if the original speech is duly maintained through content and style, the addressee is not able to transfer the entire context of the speech act.

A large degree of precision is maintained upon citation of written sources, and, even then, there is a danger of distorting the meaning of another person's words through shortening the quote or associating it with a different context.

The challenge of analysing the phenomenon of citation within a newspaper text is embodied in the fact that such a text relies simultaneously on the interpretation of the spoken and written sources, as opposed to, for example, scientific texts, where only published works are referenced, and which readers may check by themselves.

A second challenge is the commitment to a broad range of recipients: an audience with different background knowledge and experience. Thus, every recipient's understanding of the given situation may vary, which leads to uneven interpretation and nonuniform understanding and explication of the text. Without a doubt, that requires the authors of mass media texts to engage in citation being fully aware of the power of this language resource.

In our view, the primary pragmatic functions of citation in a newspaper text lie in substituting a journalist's words with words from an original source; for the purpose of illustrating a point and giving the text credibility; for argumentation and evidence of the journalist's conclusions (Terentieva, 2016).

Considering the diversity of the texts cited in mass media, it is possible to distinguish between two groups of source texts:

– texts (written or spoken expressions, documents) carrying momentary significance (the topicality of which is of a temporary nature) and those that obtain social importance only within a newspaper text; and
– precedent texts in the form of aphorisms, adages, or maxims etc. laden with a given people's generalized conceptions about the world, or in the form of utterances, referring the addressee to the texts repeatedly reproduced within the given community (Scripture, literary pieces, film names, songs, etc.).

In our view, since the research material appears as a printed text, an evident formal criterion upon initial differentiation of citation forms is the presence or absence of graphically distinct quotation marks. In connection with the aforementioned, it is viable to use the following tentative definitions in the upcoming description: *direct* and *indirect* citation.

By *direct* citation, we mean a literal reproduction of another person's speech, graphically outlined through quotations marks.

By *indirect* citation, we mean a content retelling of another person's speech without a special graphical outline through quotation marks. Above all, indirect citation is characterized by the content conveyance of another person's utterance.

In addition, we shall single out *mixed* citation as a combination of direct and indirect citation in a single headline.

The citation of any given precedent text, significant for the present linguo-cultural community, we call *implicit citation*.

The text which is formulated in a citation – is merely an aspect of the speech act, which includes various linguistic and extralinguistic elements. The author of the publication often introduces to the conveyance of another person's speech his or her personal understanding of the implicit intentions of the cited author. One element of such interpretation is the choice of the speech verb (verba dicendi), which, apart from designating the speech act itself, may also carry additional information about the disposition of the speech act (e.g., reproduce the manner of speech) or evaluate the cited words – thereby expressing the author's attitude towards the cited text or sometimes outlining the publication's own position. For this reason, the following work analyses the most frequent predicates introducing citation in the Spanish press.

### 2.3 Features of a newspaper headline

The language of mass media has long been studied by linguists (Bell, 1991; Casado Velarde, 1995; Escribano Hernández, 2006; Lázaro Carreter, 1977, 1990; Martínez Albertos, 2001; Romero Gualda, 1994; Richardson, 2007; Jaki, 2014). A newspaper text is commonly viewed as a quadruple structure, with a headline, introduction, main body and a conclusion as its primary compositional elements, inherent to any newspaper genre. In the present research, citation is analysed within the scope of Spanish press headlines. A headline of any newspaper text is of paramount communicative importance (Pou Amérigo, 2004; Casasús Guri, 1991; Gómez Sánchez and Guerra Salas, 2011; Hurtado González, 2009). Notably, Luis Pablo Francescutti (2009) reviews the dynamic in choosing headline verb forms over the span of 25 years. Miguel Ángel Rebollo Torío (2008) juxtaposes headlines from the Spanish press and a number of Latin American newspapers to study means of language. An article by María José Ruiz Acosta (1992) is devoted to historical changes, undergone by newspaper headlines in Spain as well as the particularities of their modern structure.

Based on the tradition present in the Spanish press, the headline is tasked with accomplishing three main objectives:

– to inform the reader of the content of the article;
– to convince the reader that the content shall be of interest; and
– to be a complete informative message in its own right.

Newspaper headlines are designed to arouse a reader's interest in the article that follows and to summarize its content. A substantial circumstance is that many readers limit their newspaper reading experience to scanning headlines alone, which means the latter are required to be as informative as possible. This is especially relevant for electronic mass media (Ávila, 2006).

An objective of a headline is to impact a reader in a certain way (Gómez Mompart, 1982). Lorenzo Gomis, a Spanish researcher of the newspaper style, writes, “…the objective of a headline is to spark interest, draw attention, make an impression and make sure that the reader thinks and expresses their opinion” (Gomis, 1992: 59).

Various Spanish print publications impose specific requirements on headlines. Many Spanish periodicals publish so-called *libros de estilo* (stylistic manuals), id est recommendations for their employees concerning language and style (Libro de estilo de El País, 2004; Libro de estilo de El Mundo, 1996; Libro de estilo de ABC, 2001; Libro de redacción de La Vanguardia, 2004; Berrocal Gonzalo and Rodríguez-Maribona, 1998; Santiago, 1998; López Hidalgo, 2001). The aforementioned include regulations about the headline formatting, its length, verb tense, punctuation etc. A headline is required to feature precise wording and a high degree of information value. In this work we are to examine these recommendations, most notably, with a focus on employing implicit citation in headlines.

Often, to increase the impact of the article, authors resort to citation in headlines. In the Spanish press, this is a rather widespread phenomenon. Such a technique allows the writer to make events concrete and instil extra credibility.

Of particular interest is a work by Silvia Hurtado González (2009), which follows research by Antonio López Hidalgo (2001) and outlines a detailed classification of typological features of headlines, as well as their pragmatic functions. A comparative study of Spanish and Latin American newspapers allowed the author to bring to light the frequency of the narrating remark position or lack thereof and draw conclusions about the presence of various citation forms in headlines as a reflection of the speech act. The author also singles out the hypertextual function of a headline as being inherent specifically to electronic mass media.

Spanish scholars Marina Fernández Lagunilla and Covadonga Pendones (1997) study the forms of reported speech in the headlines of the Spanish press based on the El Pais newspaper materials, distinguishing a direct, an indirect and mixed method of its introduction. Much attention is given to headline wordplay, including phenomena which in our research we refer to as implicit citation. The authors strive to discern various techniques, inherent to the informative style- or authorial style-headlines.

The most in-depth analysis of the citation phenomenon in the Spanish press is conducted by Juan Gabriel Nadal Palazón, 2008, Nadal Palazón, 2011. The author builds their research upon informative articles in the Spanish and Latin American media. Producing an all-round analysis of the material, the author defines the primary functions and objectives of the headlines, examines the verb tenses used, the syntactic structure, the elliptical constructions, etc. When describing forms of citation, Nadal Palazón deems it necessary to single out direct speech, reported speech, narrated discourse and mixed forms, describing potential varieties in detail. The selected citation classification system leads the author to the following results: media headlines are especially valuable for the study of reported speech, since it is encountered in more than 60% of them; the percentage ratio between the 4 declared citation forms is as follows: 61,2% - indirect citation, 23,6% - narrated discourse, 12% - direct citation, 3,2% - mixed forms. Therefore, from Nadal Palazón's standpoint, the Spanish-speaking world demonstrates a tendency to communicate reported speech in mass media headlines through indirect citation.

## 3 Aims and objectives

The goal of the current work is full-scale research of citation in newspaper headlines as a method of representing reported speech, based on Spanish electronic mass media. We rely on our own division of cited texts as presented above, with two groups of texts indicated and the formal criterion of direct citation being the presence of quotation marks.

Our objectives include the following:

a) defining a percentage of headlines among leading electronic mass media outlets in Spain that incorporate citation;
b) defining the varying forms of citation typical of said sources; and
c) reviewing the role of the narrating remark when reproducing reported speech within the newspaper text.

Since a modern person's experience of reading electronic mass media often consists of scanning headlines (since following the link to the full text requires additional effort), it may be assumed that authors will resort to quotations in headlines with increased regularity.

## 4 Materials and methods

### 4.1 Analysis materials

To supply our study with data, five electronic versions of leading Spanish newspapers were chosen: *El País* (established in 1976, with the electronic version issued since 1996, published daily, 1.027.000 readers every day), *El Mundo* (established in 1989, with the electronic version issued since 1995, published daily, 702.000 readers every day), *20 minutos* (established in 2000, with the electronic version issued since 2005, published Monday through Friday, 607.000 readers every day), *La Vanguardia* (established in 1881, with the electronic version issued since 1995, published daily, 572.000 readers every day), and *ABC* (established in 1881, with the electronic version issued since 1995, published daily, 408.000 readers every day) (20 minutos.es).

The following publications belong to the so-called quality Spanish press, which is characterized by.

– nationwide prevalence across all Autonomous Communities and, thus, the ability to influence the entire population of the country;
– the choice of topics, representing national interest;
– direct and continuous contact with state and governmental organizations and institutions; and
– chief editorial offices located in the capital.

The indicated criteria played a key role in selecting the materials for the study.

### 4.2 Methodology

Selected and analysed within the scope of the current research are headlines from the above publications belonging to an 8-year period. The total quantity of the analysed headlines is 1730. The selection of issues was conducted based on a chronological principle without tie-in with specific political events: a single November issue of every publication for 2010, 2012, 2014, 2016 and 2018 was selected for the study. The selection centred on a two-year interval and was carried out to achieve a more contrastive exposure of potential changes in the forms of citation.

**Figure 1:**
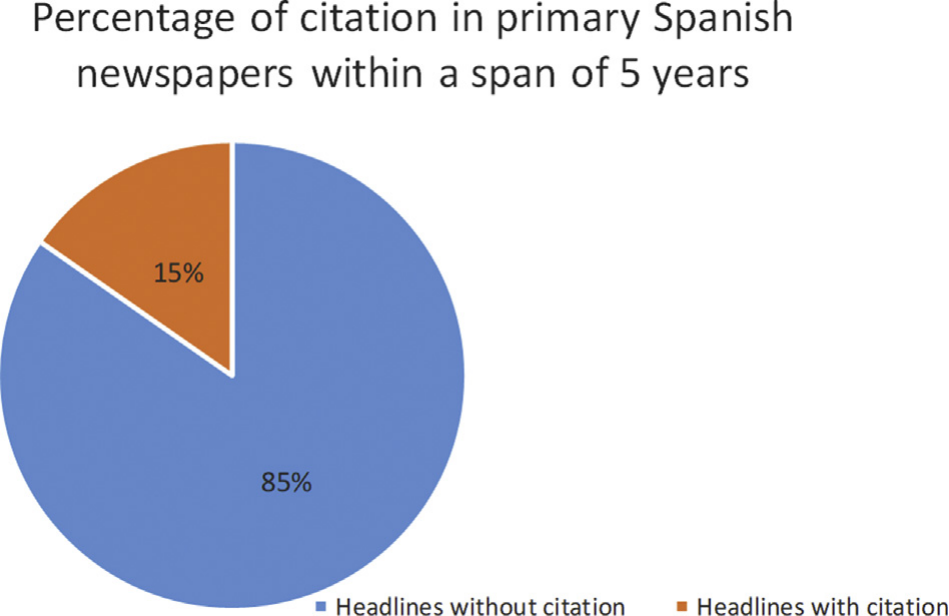
The percentage of headlines containing citation within the studied time span.

**Figure 2:**
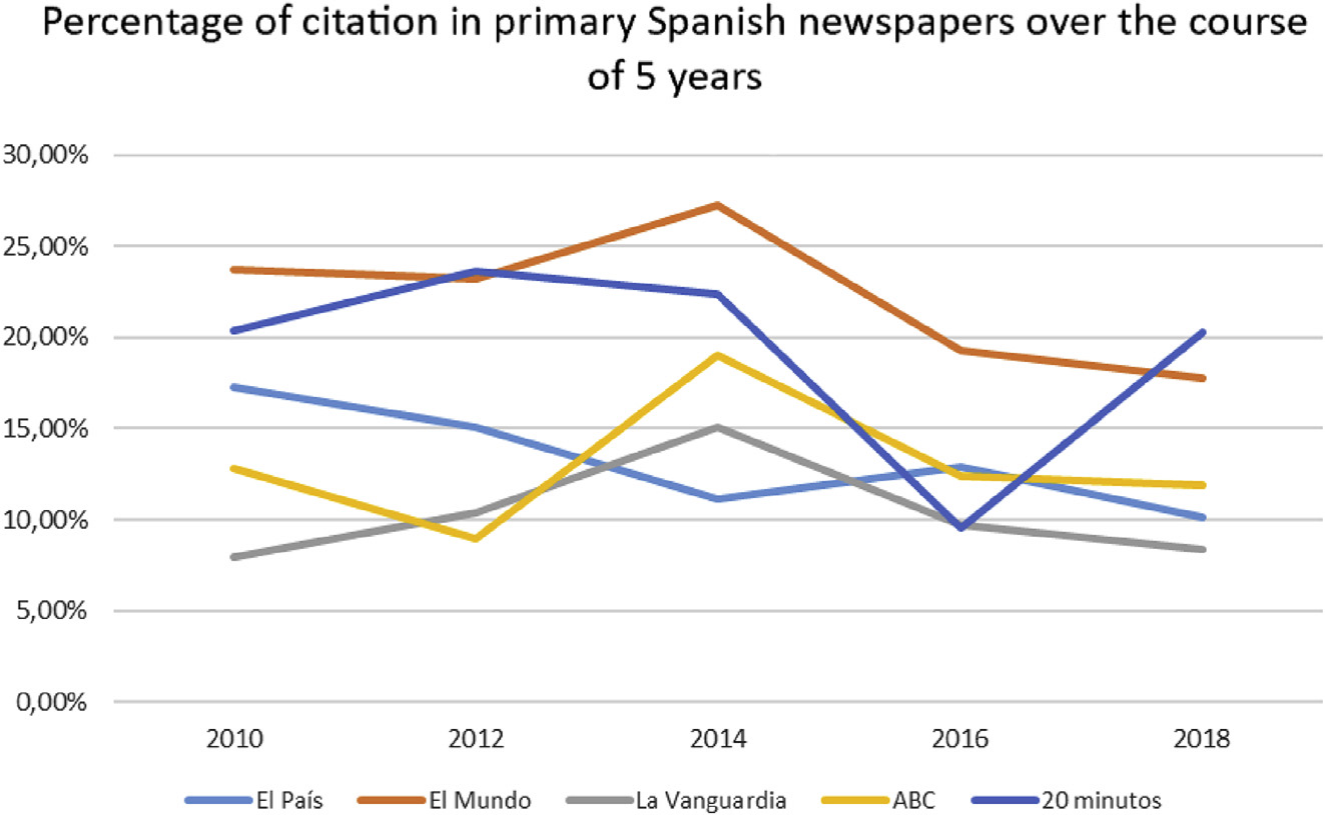
The development of the percentage ratio in headlines with citation in mass media over the course of the time span in question.

All the headlines in question have been subjected to analysis which yielded a selection of headlines with citation present in one form or another. The identified citations were divided into four groups according to the following classification: direct, indirect, mixed and implicit (see section 2.2). The next step involved converting qualitative data into a quantitative result. The conducted content-analysis presented the essence of the headlines as a numerical expression and in accordance with the stated categories. The key comparison parameter was the percentage ratio between various citation types. A total percentage of headlines with citation during the indicated period was calculated along with the change in the percentage ratio which occurred within the outlined timespan (Figures 1 and 2). Independent graphs were formed to represent direct and indirect citation throughout different years and periodicals (Figures 4 and 5). The material in question was subjected to a comparative analysis. The procedure was executed to identify the primary tendencies underlying the use of citation in quality Spanish press as well as to single out the presumed differences in approach to citation among different publications. Additionally, based on the analysis of the formed corpus of headlines, various citation-introducing methods were described. Overall, the graphs and tables were generated with consideration of the set goals and with the intention to provide the most evident demonstration of the results yielded by the conducted study.

## 5 Results

The analysis conducted within the scope of the stated methodology allowed us to formulate the following conclusions.

### 5.1 The percentage of headlines with citation

The percentage of headlines with citation within the studied time span amounts to 15% of all headline constructions (see Figure 1).

Figure 2 demonstrates how the percentage of headlines with citation in given publications evolved in the course of the time span in question.

The above indicates that the percentage of citation across headlines in the Spanish press fluctuates between 8% and 27%. As seen from the graph, even though *El Mundo* resorts to citation in headlines more frequently than other publications, we may nonetheless rely on the average data across all publications combined.

**Figure 3:**
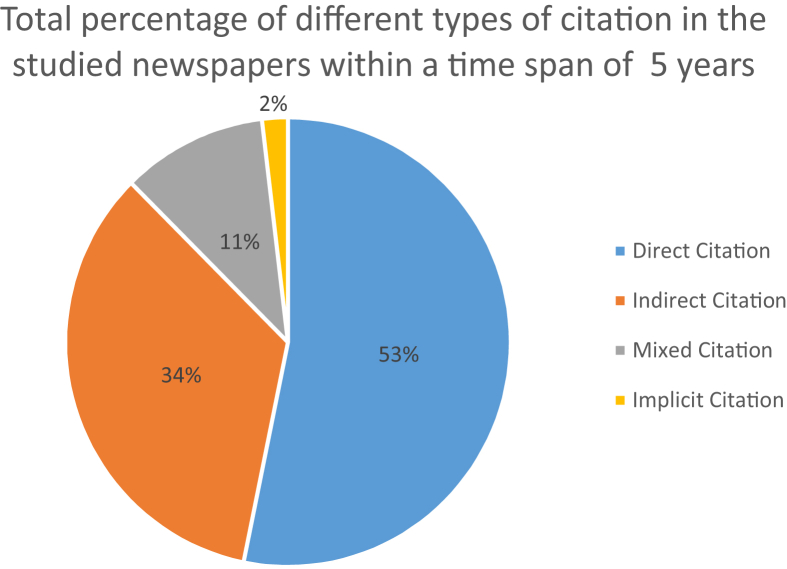
The share of citation types across headlines in mass media within the time span in question.

As stated in section 4.2, citation in a headline may be encountered in a direct, indirect, mixed or implicit form. The share of each type is outlined in Figure 3.

As illustrated by the graph, direct citation constitutes more than half of the examples in our research.

### 5.2 Direct citation in a newspaper headline

**Figure 4:**
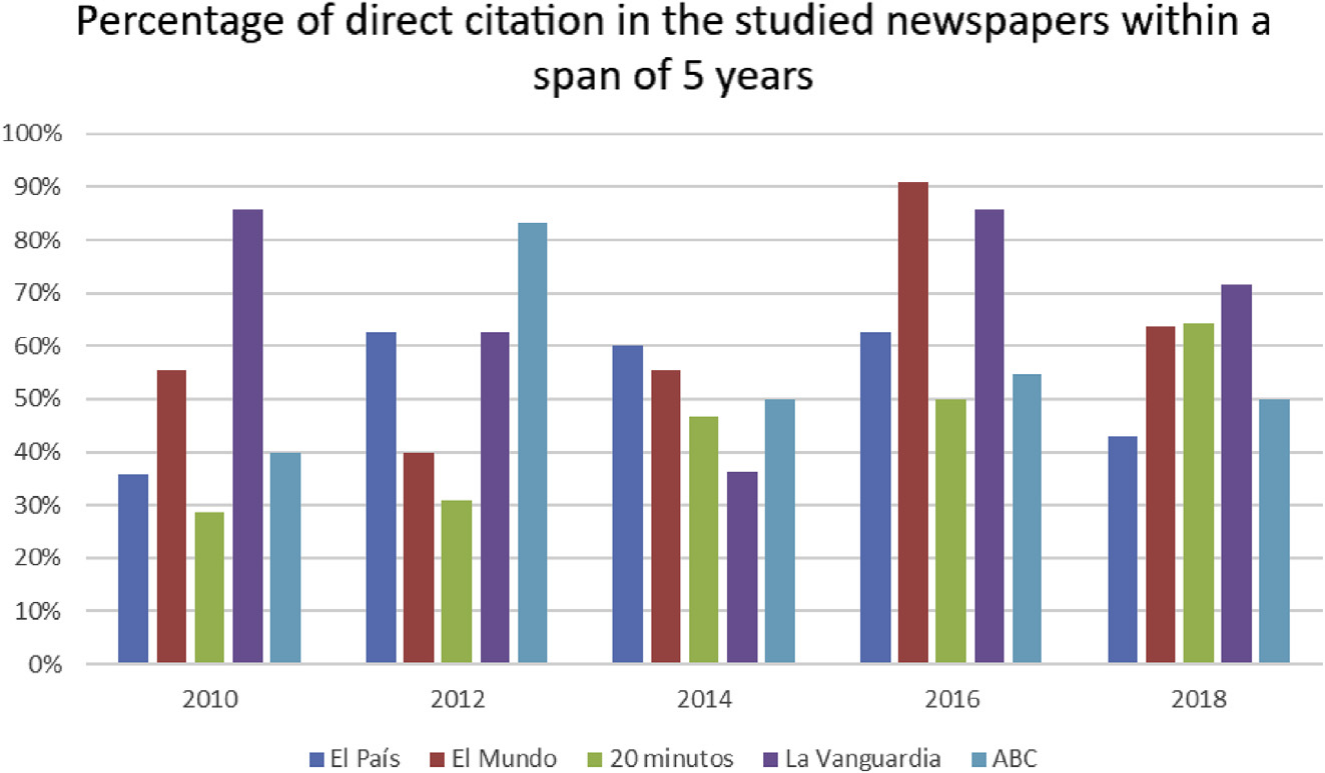
The percentage of direct citation in the total amount of citation in mass media headlines within the time span in question.

The analysis of structural specifics of direct citation (see Figure 4 in Spanish newspaper texts allows us to single out the following types: total (68%), reduced (28%) and segmented (4%) citations.

Total citation indicates the presence of a semantically complete excerpt of the source text without its abridgement in the newspaper text.

All given examples contain semantically complete statements, executed with due compliance with the rules of distinguishing direct speech in the Spanish language (colon, quotations marks, etc.), inherent in the newspaper style.

a) At times, the headline consists solely of the citation without reference to its author. In such cases, the author is usually pointed out in the subheading.

“Isabel tenía razón cuando repetía que iba a ir a la cárcel” (“Isabel was right when she said I'd end up in jail”) (ABC, 05/11/2014).

“Los hombres me dicen que no vienen obligados” (“People say they owe nothing”) (El País, 01/11/2018).

b) A fragment of the source text is supported by a narrating remark, built in accordance with rules of direct speech.

Bernie Ecclestone admite la grave crisis de la Fórmula 1: “No sé cómo arreglarlo” (Bernie Ecclestone admits Formula 1 in deep crisis: “No clue how to fix things”) (20 minutos, 02/11/2014).

Casado se distancia de Cospedal: “Mi único compromiso es con los afiliados” (Casado distances himself from Cospedal: “My responsibilities lie only with party affiliates”). (El País, 01/11/2018).

c) Fairly frequent is a technique of free-direct speech (discurso directo libre) (Maldonado González, 1991), when the headline omits a speech verb and the citation is introduced following a colon after the indication of the utterance author's name.

Valeriano Gómez: “España necesitará salarios moderados durante mucho tiempo” (“Valeriano Gómez: “Spain to limit wage level for a long time to come”) (El Mundo, 04/11/2010).

Carmen Posadas: “Me llama la atención la gente que convierte su vida en una novela por entregas” (Carmen Posadas: “I see that some people spend their lives chasing after subsidies”) (ABC, 05/11/2018).

A percentage ratio of the aforementioned types of total citation are demonstrated in Table 1.

**Table 1:** A percentage ratio of total citation types in mass media headlines within the time span in question.

Types of total citation	No utterance included, a citation without indication of the author	In accordance with the rules of direct speech	Containing an omitted speech verb. Source: “…”.
Percentage within the total amount of citation	16.23%	4.53%	15.85%

Reduced citation implies cases where an excerpt of the source text is presented in an abridged form and which may unequivocally be understood exclusively within the appropriate context. Upon reduced citation, quotation marks may be used to distinguish any length of the text with a complete or incomplete syntactic structure. Only the most information- or opinion-rich fragment of the source text is presented, along with a technique of pseudo-direct speech (*discurso pseudo-directo*) (Maldonado González, 1991). In this case, the citation has to be naturally integrated in the text.

Argentina suspende el contrato de Talgo por coste “excesivo” (Argentina suspends Talgo's contract due to “exorbitant” financial demands) (El País, 03/11/2012).

El accidente de la nave Virgin Galactic, “triste pero predecible” (Virgin Galactic ship accident “sad but predictable”) (El Mundo, 01/11/2014).

Segmented citation is a type of direct citation represented by a sequence of semantically interconnected citation inserts from the source text, introduced at intervals.

Una bola de fuego “más brillante que la Luna” convirtió “la noche en día” en el sureste de España (Fireball “brighter than the Moon” turned “night into day” in the South-East of Spain) (20 minutos, 03/11/2018).

La Fiscalía abre expediente para “proteger” a la niña madre y “no para perseguir delitos” (Prosecutor's Office commences action to “protect” underage mother, “not to fight crime”) (El Mundo, 04/11/2010).

Direct citation provides the effect of maximum engagement for the reader, “removing” the journalist from the informational delivery and illustrating the author's words.

### 5.3 Indirect citation in a newspaper headline

**Figure 5:**
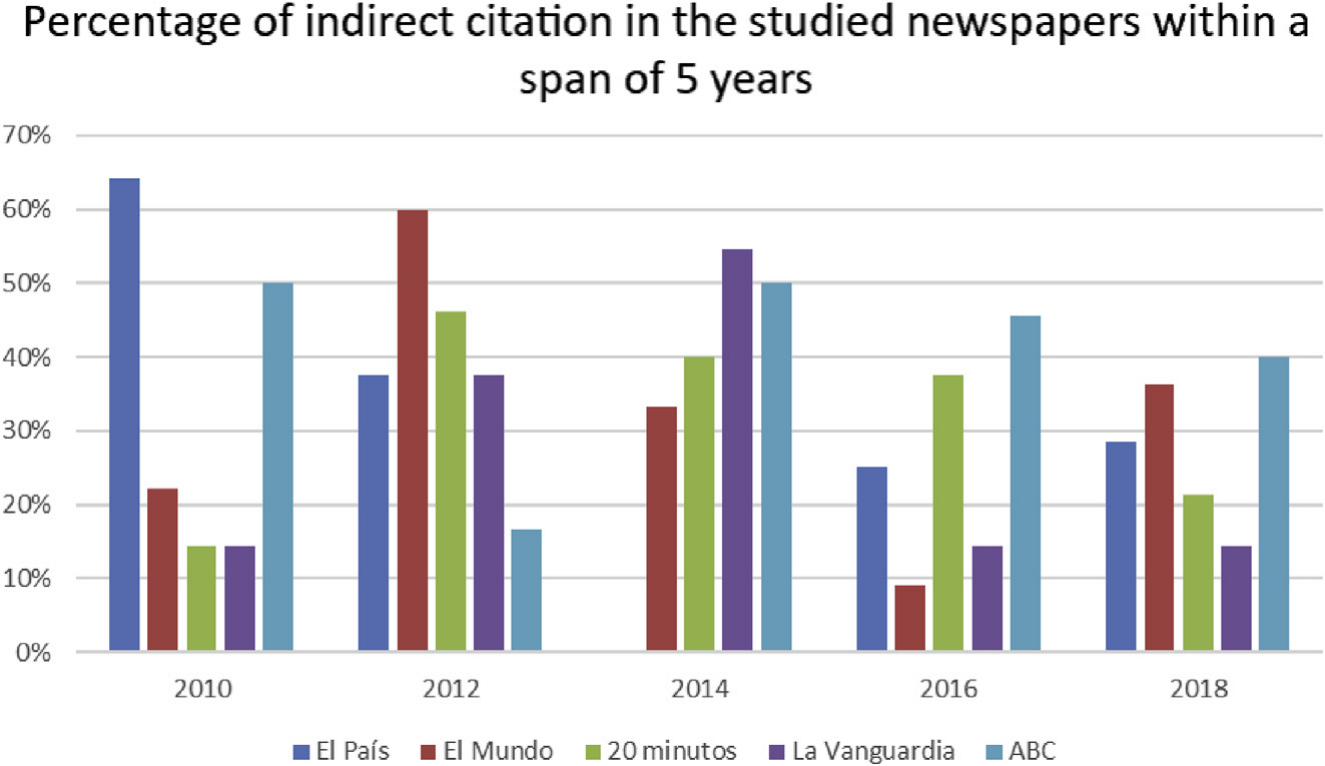
The share of indirect citation in the total amount of citation in mass media headlines within the time span in question.

Less frequently, a newspaper headline will contain indirect citation (see Figure 5).

Indirect citation may be expressed through the following main use scenarios.

a) A rendering of an indirect speech pattern (with or without the sequence of tenses, an inherent feature of a newspaper text).

Dirigentes del PP reclaman que Rajoy expulse a todos los cargos imputados (The People's Party leadership demands Rajoy to oust everyone involved in the matter) (La Vanguardia, 03/11/2014).

El contable de Correa dice que nunca pagó dinero a políticos (Accountant Correa maintains he never paid politicians off) (ABC, 01/11/2016).

b) The use of the quasi-indirect speech technique (*estilo casi indirecto*), whereby a journalist passes a message as his or her own speech while simultaneously referencing the source of information (Reyes, 1994: 20). A retelling with an indication of the information source of this kind is typical of the Spanish press. Journalists build a message as their own speech, and yet, to charge their message with more trustworthiness and to obscure their own responsibility for its content, they state the information source. With that, the newspaper text is not made bulky through heavy syntactic structures; at the same time, the essence of the conveyed information becomes central – but not the history of its reception. This technique, however, still allows for maintaining distance between the citing text and the source text.

Similar functions are fulfilled by the *según* preposition, which references the source of information.

Políticos franceses de todo signo critican la entrega a España de Aurore Martin (French politicians of all breeds criticise the extradition of Aurore Martin to Spain) (El Mundo, 03/11/2012).

Zajárchenko gana en la autoproclamada república de Donetsk, según sondeo (Polls show Zakharchenko's victory in the self-declared Donetsk Republic) (20 minutos, 02/11/2014).

The percentages of the aforementioned types of introducing indirect citation are shown in Table 2.

**Table 2:** The share of indirect citation types in mass media headlines within the time span in question.

Types of indirect citation	In accordance with the rules of indirect speech	Quasi-indirect speech: *estilo casi indirecto*
Percentage within the total amount of citation	14%	20%

Indirect citation allows to juxtapose certain utterances with a shorter narrative – to express the essence of another person's utterance.

### 5.4 Mixed citation in a newspaper headline

At times, indirect speech blends with separate elements of direct citation. This would be the same known phenomenon of pseudo-direct speech.

The following type of headlines can be divided into two groups:

а) reported speech pattern with reduced or segmented direct citation;

El cardenal de Bagdad afirma que “no tememos a la muerte” (Bagdad cardinal claims, “we are not afraid of death”) (ABC, 03/11/2010).

Blanco dice que Feijóo “pone la firma” y él “la pasta”, y cree que Galicia “no necesita amigos como Rajoy” (Blanco says Feijóo “puts signatures” while he “puts money on the table” and claims that Galicia “has no need of such friends as Rajoy”) (20 minutos, 07/11/2010).

b) quasi-indirect speech with reduced or segmented direct citation.

Merkel pide un “gran esfuerzo” durante otros cinco años para superar la crisis (Merkel asks to make “a great effort” for five more years to overcome crisis) (El Mundo, 03/11/2012).

Xulio Ferreiro recuerda a los que “lucharon” por una ciudad “más justa” en una ofrenda en San Amaro (At San Amaro commemorative ceremony Xulio Ferreiro remembers those who “fought” for “a fairer” city) (20 minutos, 01/11/2016).

The percentages of the aforementioned types of introducing mixed citation are shown in Table 3.

**Table 3:** The share of mixed citation types in mass media headlines within the time span in question.

Types of mixed citation	Reported speech pattern + reduced or segmented direct citation	Quasi-indirect speech + reduced or segmented direct citation
Percentage within the total amount of citation	4%	7%

On the one hand, mixed citation allows to maintain the essence of the information in a concise form, while on the other hand it allows to retain a reader's attention through the inclusion of direct citation.

### 5.5 Implicit citation in a newspaper headline

We use *implicit citation* as a term in the footsteps of Anna Wierzbicka (1970). They are a kind of “insert” or “alien bodies” in the text, a citation of precedent texts.

The term “precedent text” was once proposed by Yuri Karaulov (1986), who used it to define texts meaningful for a person from informative or emotional standpoints, and which are well known in the milieu of that person (including his or her contemporaries and predecessors); the discourse of the said language persona involves repeated references to those texts.

Being a representative of a national-linguo-cultural community, a language persona commands the cognitive base of the said community out of necessity, id est the totality of knowledge and notions possessed by all natives of a national-linguo-cultural mentality – all speakers of a language. Precedent phenomena belong to the primary elements of a cognitive base.

Precedent texts are included in the foundation of historic memory of a linguo-cultural community; they make up a part of its background knowledge and belong to cultural concepts.

Victoria Krasnykh points out that for successful, adequate communication to take place – one that achieves a more or less complete but always sufficient mutual understanding from the communicants’ point of view – a degree of shared knowledge is a necessary requirement. Otherwise, there is a possibility of a “communicative glitch (insufficiently adequate communication, insufficiently complete mutual understanding of the communication participants) or a communicative failure (inadequate communication, complete lack of understanding between the communicants)” (Krasnykh, 1998: 169).

Implicit citation may be used within the newspaper text itself or its headline. Here are the encountered examples.

Lavadoras con muerte anunciada (Washing machines with planned obsolescence) (El País, 02/11/2014). The headline of the article about outdated machinery alludes to a novel by the famous Colombian writer Gabriel García Márquez, “Crónica de una muerte anunciada” (“Chronicle of a Death Foretold”).

‘OT 2018’ gala 6: Lo hacemos y ya vemos («Operation Triumph, 2018» gala 6: Let's do it and then we will see) (El País, 02/11/2014). The headline of the article about the backstage affairs of a popular TV show contains a reference to a song “Lo Hacemos y Ya Vemos (De "La Llamada")” (“Let's do it and then we will see (from "La Llamada" – “Holy Camp!”)”) by Anna Castillo featuring Macarena García.

Mucho juego para tan poco oficio (Much ado about nothing) (La Vanguardia, 03/11/2014). The headline of the article about a soccer match employs the modified proverb “mucho pollo para tan poco arroz” (“many chickens, little rice”), roughly translated as “not worth a whistle”.

Doble impacto para los empleados (Double impact for employees) (La Vanguardia, 04/11/2018). The precedent text in the headline of an article about issues in the banking sector is the name of the famous film “Double Impact”, starring Jean-Claude Van Damme.

¿Quién teme a la Guardia civil? (Who's afraid of the Civil Guard?) (20 minutos, 05/11/2018). The headline of an article about the work of the Civil Guard alludes to a play by American playwright Edward Albee, “Who's Afraid of Virginia Woolf?”, the name of which, in turn, relates to a famous song from the fairy tale “Who's Afraid of the Big Bad Wolf?”

A transformation of precedent utterances of this kind on the pages of central Spanish press is rather infrequent. The stylistic manuals, published by leading Spanish publishers, often condemn using unchanged or transformed names of literary pieces, films or songs in newspaper headlines. The headlines of the following type are seen as a sign of “lacking imagination and laziness of the mind” on the journalist's part (Libro de estilo de El País, 2004: 54). “It is not advised to partake in excessive originality,” write the authors of the La Vanguardia (2004: 61) stylistic manual, since from their point of view – journalists keep rephrasing the same sources (namely, the names of novels by Gabriel García Márquez “Chronicle of a Death Foretold” and “No One Writes to the Colonel”, as well as plays “A Streetcar Named Desire” written by Tennessee Williams and “Six Characters in Search of an Author” by the Italian playwright Luigi Pirandello). A clear, informative headline is of highest priority. In his educational materials “El estilo del periodista” (“A journalist's style”), Álex Grijelmo also warns against « refritar una idea que ya circula» (“a repeated warmup of an already established idea”) (2003: 477). An attempt to be original leads to the exact opposite: a banal recycling of the same known phrases, whereas an author's goal is to construct their own headline.

The adherence to the above rules by the journalists affiliated with the leading Spanish publications is also proved by the figures in our study (see Table 4).

**Table 4:** Occurrence of headlines containing implicit citation, in numbers.

	El País	El Mundo	20 minutos	La Vanguardia	ABC
2010	0	0	0	0	0
2012	0	0	0	0	0
2014	**1**	0	0	**1**	0
2016	0	0	0	0	0
2018	**1**	0	0	**1**	**1**

### 5.6 The role of a narrating remark

Also worth contemplating is another element related to the pragmatic aspect of citation – a *narrating remark*. The character and pragmatic orientation of citation are largely defined by the content of the remark that accompanies the citation. Speech verbs, or verba dicendi, express human vocal acts carrying a communicative vector. There are multiple classifications of speech verbs (Paducheva, 2004; Searle, 1976; Maldonado González, 1991; Reyes, 1993).

As far as the Spanish newspaper text is concerned, we believe that singling out three main predicate groups introducing direct and indirect citation will be more informative: *declarative*, *explicative* and *evaluative* predicates. Such a division is relative, as many predicates that introduce citation also perform an integration function along with a number of other specific functions, such as the evaluation of citation content, providing commentary of varying sorts, concretization, addition, etc.

Thus, we distinguish between the following main types of predicates fulfilling the role of narrating remarks in a Spanish newspaper text:

1. Declarative predicates (*decir –* to say*, anunciar –* to announce*, considerar –* to consider*, constatar* – to note*, manifestar* – to state*, describir* – to describe*, mencionar* – to mention, *contar* – to tell*, relatar* – to narrate*, declarar -* to declare*, indicar* – to indicate*, señalar –* to point*,* etc.).

To this group we assign predicates containing, above all, an indication of the fact of the speech event; although, as mentioned above, every citation-introducing verb (except for *decir*) contains additional semantic components.

The Spanish press is notable for a substantial diversity of declarative verbs introducing direct and indirect citation, which is due to the objective of the newspaper text itself (the conveyance of specific information) as well as due to stylistic requirements.

Irán anuncia el arresto de cuatro británicos relacionados con actos terroristas (Iran declares four British arrested in connection with terrorist attacks) (El Mundo, 04/11/2010).

El Banco de España dice que la subida del salario mínimo costará 150.000 empleos (Bank of Spain says increasing minimal wage to cause 150 000 redundancies) (El País, 01/11/2018).

2. Explicative predicates (*pedir* – to inquire*, exigir* – to demand*, insistir* – to insist*, concluir –* to conclude*, contestar -* to answer*, responder –* to reply*, exclamar –* to exclaim*, preguntar* – to ask*, resumir –* to summarize*, agregar* – to add*, explicar –* to explain*, continuar –* to continue*, enfatizar –* to emphasize*, puntualizar –* to point out*, subrayar –* to underline*, concretar* – to specify*, precisar –* to precise*, repetir –* to repeat*,* etc.).

This group is constituted by predicates oriented at recreating the character of a speech event.

On the account of the fact that the newspaper text often cites spoken statements, the choice of a citation-introducing verb is to compensate for the lack of an addressee during the speech act – to explain a wider context of the initial text creation. The facts of speech and words are to be truthful and the additional elements (the character of the speech act, the manner of speech, the emotions) are conveyed exclusively through the interpreter and, among other things, through the choice of the introducing predicate. Such additional elements serve to actualize, create an imitation of natural dialogic speech (*replied, repeated, added*) to establish a conversation partner presence-effect during the creation of the source text; this leads to the “theatricalization” of speech, an active involvement of “somebody else” in the created text.

Pablo Iglesias pide a ERC y PDeCAT que apoyen los presupuestos para que “no pague la gente” (Pablo Iglesias asks left-wing Republicans and Catalan Dems to support the passing of the budget, “not to make people pay”) (20 minutos, 03/11/2018).

El Papa insiste, al regreso de Suecia, en rechazar el sacerdocio femenino (Upon return from Sweden, the Pope still insists women cannot be priests) (La Vanguardia, 02/11/2016).

3. Evaluative predicates (*criticar* – to criticize*, denunciar –* to refute*, reclamar –* to claim*, descartar* – to deny*, asegurar* – to ensure*, calificar –* to qualify*, admitir –* to admit*, respaldar –* to support*, reconocer –* to recognize*, aprobar –* to approve, *alabar –* to praise, *pronosticar* – to prognosticate, *predecir* - to predict*, avisar* – to notify*, acusar* – to accuse*,* etc.).

The following group of predicates is made up of verbs and verb phrases containing a certain evaluation of the speech event.

That said, it is not always simple to identify the party evaluating the given information: the subject of the speech act or its interpreter.

La CE descarta una tercera recesión en Europa pese a empeorar sus previsiones (The European Commission denies third recession in Europe despite worsening forecasts) (ABC, 05/11/2014).

Los alcaldes afectados por la riada critican la tardanza y el reparto de las ayudas (Mayors of impacted localities criticise delay and assignment of support) (El Mundo, 03/11/2012).

**Figure 6:**
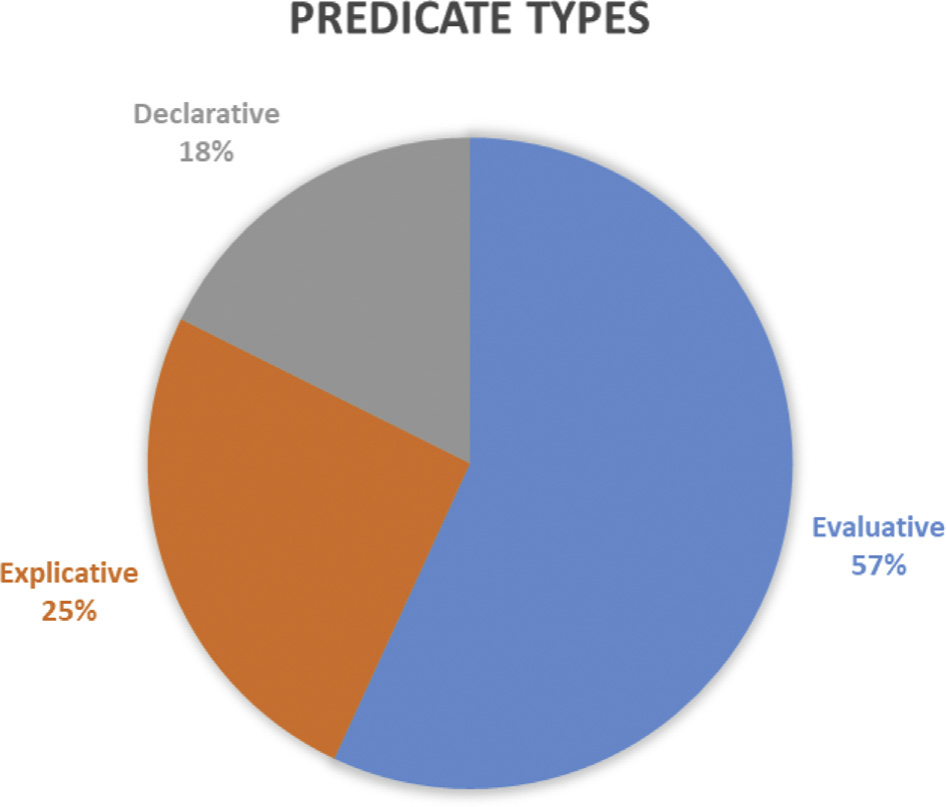
Types of predicates in mass media headlines within the time span in question.

The percentages of the three types of predicates are reflected in Figure 6.

**Figure 7:**
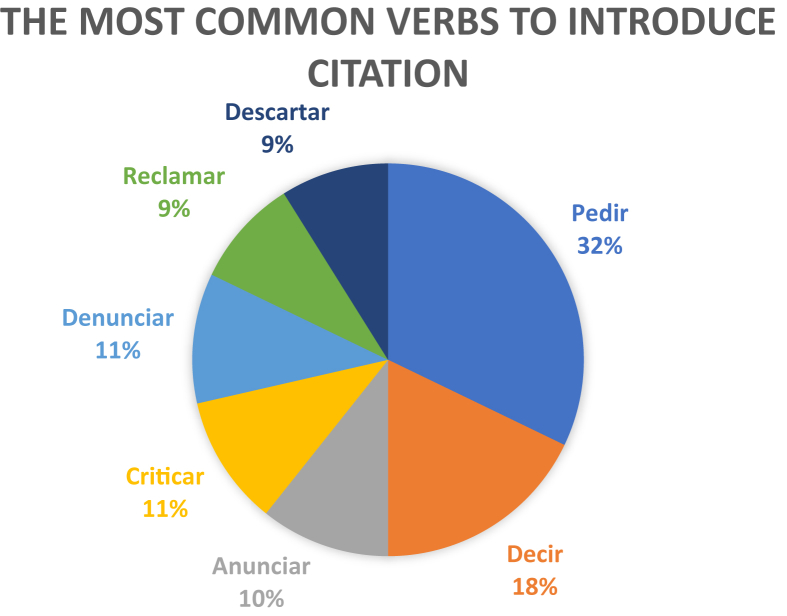
The most common verbs to introduce citation in mass media headlines within the time span in question.

In the next graph, one may observe the most common verbs to be found in the mass media headlines included in our study (see Figure 7).

## 6 Discussion and conclusions

The conveyance of information as the fulfilment of the main function of mass communication is largely accompanied by a direct or indirect expression of an evaluation, which invites the audience to a certain reaction regarding the conveyed information. Citation in article headlines plays a major role in fulfilling that goal.

As a result of the conducted work, we conclude that headlines with citation comprise approximately 15% of the total number of headlines in Spanish electronic mass media. The research demonstrates that this figure is, on average, representative of any publication within the course of the studied time spans. The presence of many headlines with direct and indirect citation confirms journalists’ aspiration to make headlines as informative and compelling as possible.

The chosen formal criterion (the presence or absence of graphically distinct quotation marks) allowed us to distinguish direct (53 %), indirect (34 %) and mixed (11 %) citations. Therefore, according to our calculations, quotation marks in headlines are used in more than 60% of inclusions of reported speech. This data differs from conclusions drawn by Juan Gabriel Nadal Palazón (2008, 2011) about the prevalence of various forms of indirect citation in the Spanish press headlines. This is due to the author's broad understanding of the citation factor. By contrast, our research (based on a formal representation of direct citation as a fragment of reported speech in a text marked by quotation marks) – indicates that most of the studied material is represented by direct (total, reduced or segmented) or mixed citation. It is also important to consider that Nadal Palazón's research relies on the materials of Latin American newspapers.

Alongside with describing direct, indirect and mixed citation, we found it necessary to analyse the narrating remark, which is tightly knit with the pragmatic aspect of citation. This topic became the object of study, inter alia, in a research by Silvia Hurtado González (2009), where citation-introducing speech verbs are analysed based on the formal criteria of their position within the headline (pre-position, post-position in relation to the quote, or total lack thereof). Our research studies speech verbs based on their aptitude for completing specific pragmatic objectives. We distinguish three groups of predicates: evaluative (57 %), explicative (25 %) and declarative (18 %). The choice of a speech verb determines whether a journalist distinguishes the fact of the speech event itself or interprets it, thus creating the illusion of dialogic speech or introducing a certain evaluation of the speech event. This confirms that upon opting for the *de dicto* strategy, the speaker, while describing a speech act performed by another person, aspires to convey the evaluation of the speech act subject with as much precision as can be afforded, to convey the chosen nominations, the immediate illocutionary goal, etc. However, often the speaker may not report impartially and instead choose to weave into reported speech his or her own interpretation of the hidden intentions of the speech act subject, to give an evaluation of the aptness of the said speech act or its content; in other words, they choose the *de re* strategy (Shmelyov, 2002; Escribano Hernández, 2007). Indubitably, practically any citation is a kind of reflection of another text and may not be fully identical to the initial model.

We identified two groups of sources for citation which allowed us to select and attribute implicit citation (citation of precedent texts) to a separate group. Analysis shows that citation of this kind is a rare occurrence in the Spanish press, accounting for about 2% of the entire studied corpus of headlines with citation. In this regard, material which we found particularly interesting was a research by Marina Fernández Lagunilla and Covadonga Pendones (1997), conducted based on the materials of the El País newspaper, where similar cases are overviewed within the scope of analysing headline clichés and wordplay.

The analysis demonstrates that citation in headlines is an important and relevant resource for the emerging language of mass media outlets, and approximately 1/6 of headlines of the leading electronic quality press outlets use it. The study of the citation typology showed that in order for a newspaper headline to fulfil its function – to convey the topic of the main information in a concise format and to invite the audience to read the material – citation appears to be one of the most precise and effective instruments. It would be of great interest to research the pragmatic function of citation in mass media. Such a study could be beneficial for a more in-depth analysis of this function and the notion of reported speech in mass media headlines in different languages, as well as for the creation of internal policies within print press outlets for the purposes of practical education.

## Declarations

### Author contribution statement

Ekaterina Terentieva: Conceived and designed the experiments; Performed the experiments; Wrote the paper.

Galina Khimich: Conceived and designed the experiments; Wrote the paper.

Irina Veselova: Analyzed and interpreted the data; Wrote the paper.

### Funding statement

The publication has been prepared with the support of the “RUDN University Program 5–100”.

### Competing interest statement

The authors declare no conflict of interest.

### Additional information

No additional information is available for this paper.

## References

[bib1] Arutyunova N.D. (1982). The Linguistic Issues of Reference. The New in International Linguistics. Issue 13. Logic and Linguistics (The Problems of Reference). Moscow.

[bib2] Arutyunova N.D., Arutyunova N.D., Bulygina T.V., Kibrik A.A. (1992). Speech-behavioural acts as reflected by another person’s speech. The Human Factor in Language: Communication, Modality, Deixis.

[bib3] Ávila R. (2006). De la imprenta a la internet: la lengua española y los medios de comunicación masiva.

[bib4] Bakhtin M.M. (1984).

[bib5] Bell A. (1991). The Language of the News Media.

[bib6] Beltrán Almería L. (1990). El Discurso Ajeno: Panorama Crítico.

[bib7] Benveniste E. (1966). Problèmes de linguistique générale.

[bib8] Berrocal Gonzalo S., Rodríguez-Maribona C. (1998). Análisis Básico de la Prensa Diaria. Manual para aprender a leer periódicos.

[bib9] Bruña Cuevas M. (1990). Sobre la reproducción del discurso. Philologia Hispalensis.

[bib10] Bruña Cuevas M. (1993). El discurso indirecto en periódicos franceses y españoles. Estudios pragmáticos: lenguaje y medios de comunicación, Grupo Andaluz de Pragmática.

[bib11] Cappelen H., Lepore E. (2005). Varieties of quotation revisited. Belg. J. Linguist..

[bib12] Casado Velarde M., hoy M., Seco y G., Salvador (1995). El lenguaje de los medios de comunicación. La Lengua Española.

[bib13] Casasús Guri J.M. (1991). Soluciones pragmáticas en la reducción de títulos de portada. Comunicación Soc..

[bib14] Clayman S.E. (1990).

[bib15] Davidson D. (2001). Inquiries into Truth and Interpretation: Philosophical Essays.

[bib17] Ducrot O. (1984). Le dire et le dit.

[bib18] Escribano Hernández A. (2006). Comentario de textos periodísticos: informativos, interpretativos y de opinión.

[bib19] Escribano Hernández A. (2007). El discurso reproducido en las informaciones electorales. Anàlisi.

[bib20] Fernández Lagunilla M., Pendones C. (1997). Discurso reproducido y juegos de palabras en los títulos de prensa. Discurso.

[bib21] Francescutti L.P. (2009). El tiempo de los titulares. Un análisis verbal de la titulación en la prensa española durante el periodo 1980/2005.

[bib22] Gómez Mompart J.L. (1982).

[bib23] Gómez Sánchez M.E., Guerra Salas L. (2011). Conflictos y soluciones en los titulares de prensa hispanos: clasificación y análisis. Espanol Actual.

[bib24] Gomis L. (1992). Los titulares en prensa. Origen, objetivos y funciones. Estudios de Periodística.

[bib25] Grijelmo A. (2003). El estilo del periodista.

[bib26] Harry J.C. (2013). Journalistic quotation: reported speech in newspapers from a semiotic-linguistic perspective. Journalism.

[bib27] Hurtado González S. (2009). Algunas peculiaridades de los titulares de actos de habla en la prensa española e hispanoamericana. ZER.

[bib28] Jaki S. (2014). Phraseological Substitutions in Newspaper Headlines. “More than Meats the Eye”.

[bib29] Karaulov Yu.N. (1986). The Role of Precedent Texts in the Structure and Functioning of Language Persona. Scholarly Directions and New Direction in Teaching Russian and Literature. Col. Of Reports. Moscow.

[bib30] Krasnykh V.V. (1998). Virtual reality of real virtuality? (Mankind. Conscience. Communication).

[bib31] Lázaro Carreter F., Lázaro Carreter F. (1977). El lenguaje periodístico, entre el literario, el administrativo yel vulgar. Lenguaje en periodismo escrito.

[bib32] Lázaro Carreter F., García P., Gómez A. (1990). El idioma del periodismo, ¿lengua especial? El idioma español en las agencias de prensa.

[bib33] Libro de estilo de A.B.C. (2001).

[bib34] Libro de estilo de El Mundo (1996).

[bib16] Libro de estilo de El País (2004).

[bib35] Libro de redacción de La Vanguardia (2004).

[bib36] López Hidalgo A. (2001). El titular. Manual de titulación periodística.

[bib37] Maldonado González M.C. (1991). Discurso Directo Y Discurso indirecto. Madrid: Taurus Universitaria.

[bib38] Martínez Albertos J.L. (2001). Curso general de redacción periodística. Lenguaje, estilos y géneros periodísticos en prensa, radio, televisión y cine.

[bib39] Méndez García de Paredes E. (1999). Análisis de la reproducción del discurso ajeno en los textos periodísticos. Pragmalinguistica.

[bib40] Méndez García de Paredes E., de Bustos Tovar J.J., Charaudeau P., Girón Alconchel J.L. (2000).

[bib41] Méndez García de Paredes E. (2000). La literalidad de la cita en los textos periodísticos. Rev. Esp. Linguist..

[bib42] Myasnikov I. Yu. (2008). The communicative modelling of a periodical: at the crossroads of structures. Tomsk State University Vyestnik. Philology.

[bib43] Nadal Palazón J.G. (2008). El discurso ajeno en los titulares periodísticos. Acta Poética.

[bib44] Nadal Palazón J.G. (2011). El discurso ajeno en los titulares periodísticos. Tesis doctoral.

[bib45] Paducheva E.V. (2004). Dynamic Models in the Semantics of Lexis.

[bib46] Pou Amérigo M.J., Cantavela J., Serrano J.F. (2004). Los titulares periodísticos. Redacción para periodistas: informar e interpretar.

[bib47] Reah D. (2002). The Language of Newspapers.

[bib48] Rebollo Torío M.Á. (2008). Análisis de titulares en la prensa hispana. Anu. Estud. Filol..

[bib49] Reyes G. (1993). Los procedimientos de cita: estilo directo y estilo indirecto. Madrid, Arco Libros.

[bib50] Reyes G. (1994). Los procedimientos de cita: citas encubiertas y ecos. Madrid: Arco Libros (Cuadernos de Lengua Española, O).

[bib51] Richardson J.E. (2007). Analysing Newspapers: an Approach from Critical Discourse Analysis.

[bib52] Romero Gualda M.V. (1994). El español en los medios de comunicación.

[bib53] Ruiz Acosta M.J. (1992). Los titulares en prensa: estudio de su evolución y lenguaje. Lenguaje informativo y filmográfico.

[bib54] Santiago R. (1998). Ortografía, libros de estilo y prensa diaria: el País, El Mundo y ABC. Espanol Actual.

[bib55] Sapir E. (1993). Selected Works on Linguistics and Cultural Studies.

[bib56] Searle J.R. (1976). The classification of illocutionary acts. Lang. Soc..

[bib57] Shmelyov A.D. (2002). The Russian Language and the Extralinguistic Reality.

[bib58] Shpilnaya N.N. (2014). Principles of derivational modelling of text production. Questions of Constructive Linguistics.

[bib59] Smirnova A.V. (2009). Reported speech as an element of argumentative newspaper discourse. Discourse Commun..

[bib60] Terentieva E. (2016). Pragmatic aspects of quotation in Spanish media texts. Russian Journal of Linguistics.

[bib61] Van Dijk T.A. (1977). Text and Context. Explorations in the Semantics and Pragmatics of Discourse. Longman, London.

[bib62] Van Dijk T.A. (2003). Ideología Y Discurso.

[bib63] Van Dijk T.A. (2006). Discurso y manipulación: discusión teórica y algunas aplicaciones. Trad. Marianne Peronard. Signos.

[bib64] Vicente Mateu J.A. (2007). Discurso reproducido e interpretación de la fuerza ilocutiva en la prensa escrita. Revista de Investigación Lingüística.

[bib65] Voloshinov V.N. (1973). Marxism and the Philosophy of Language.

[bib66] Wierzbicka A. (1970). Descriptions or Quotations? Sign, Language, Culture, T. I.

[bib67] 20 minutos (2018). es. 20 minutos, el periódico más leído en Madrid y el tercero en España. https://www.20minutos.es/noticia/3512047/0/egm-20minutos-primer-diario-tercero-espana/.

